# Phosphoproteomics Analysis Reveals a Pivotal Mechanism Related to Amino Acid Signals in Goat Fetal Fibroblast

**DOI:** 10.3389/fvets.2021.685548

**Published:** 2021-08-03

**Authors:** Xu Zheng, Huimin Su, Liping Wang, Ruiyuan Yao, Yuze Ma, Linfeng Bai, Yanfeng Wang, Xudong Guo, Zhigang Wang

**Affiliations:** ^1^State Key Laboratory of Reproductive Regulation and Breeding of Grassland Livestock, School of Life Sciences, Inner Mongolia University, Hohhot, China; ^2^Clinical Laboratory, The Hulunbuir People's Hospital, Hailar, China

**Keywords:** phosphoproteome, amino acid, functional analysis, motif, protein interaction

## Abstract

In addition to serving as the building blocks for protein synthesis, amino acids serve as critical signaling molecules in cells. However, the mechanism through which amino acid signals are sensed in cells is not yet fully understood. This study examined differences in the phosphorylation levels of proteins in response to amino acid signals in Cashmere goat fetal fibroblasts (GFb). Amino acid deficiency was found to induce autophagy and attenuate mammalian/mechanistic target of rapamycin complex (mTORC1)/Unc-51-like autophagy activating kinase 1 (ULK1) signaling in GFb cells. A total of 144 phosphosites on 102 proteins positively associated with amino acid signaling were screened using phosphorylation-based proteomics analysis. The mitogen-activated protein kinase (MAPK) signaling pathway was found to play a potentially important role in the interaction network involved in the response to amino acid signals, according to gene ontology (GO) and Kyoto Encyclopedia of Genes and Genomes (KEGG) analysis, and MAPK1/3 may serve as a central hub for the entire network. Motif analysis identified three master motifs, xxx_S_Pxx, xxx_S_xxE, and xxx_S_xDx, which were centered on those phosphosites at which phosphorylation was positively regulated by amino acid signaling. Additionally, the phosphorylation levels of three membrane proteins, the zinc transporter SLC39A7, the sodium-dependent neutral amino acid transporters SLC1A5 and SLC38A7, and three translation initiation factors, eukaryotic initiation factor (eIF)5B, eIF4G, and eIF3C, were positively regulated by amino acid signals. These pivotal proteins were added to currently known signaling pathways to generate a novel model of the network pathways associated with amino acid signals. Finally, the phosphorylation levels of threonine 203 and tyrosine 205 on MAPK3 in response to amino acid signals were examined by western blot analysis, and the results were consistent with the data from the phosphoproteomics analysis. The findings of this study provide new evidence and insights into the precise mechanism through which amino acid signals are sensed and conducted in Cashmere goat fetal fibroblasts.

## Introduction

Amino acids are the basic building blocks of protein synthesis, serve as precursors for certain hormones, and are important energy suppliers in cells. Amino acids also play vital roles in intracellular signal transduction and metabolic regulation, acting as signaling molecules (1). In addition to serving as a nutrient source to meet the needs for cellular growth and development, methionine can improve the reproductive performance and health status of dairy cows (2), and leucine and phenylalanine can affect the pancreatic growth and development of Holstein calves (3). Limiting amino acids are known to play important roles in mammalian growth and metabolism (4). As a primary provider of methyl groups, methionine is believed to be an important limiting amino acid in growing bulls (5), and evidence has shown that differences in species, diet composition, and growth stages are characterized by different patterns of limiting amino acids (6, 7). Compared with studies performed in cows, fewer reports have been published examining the regulatory functions of amino acids on the growth and metabolism of goats or sheep. Methionine, threonine, arginine, tryptophan, and valine have been shown to be limiting amino acids in growing lambs (8), and the intestinal availability of methionine has been shown to enhance the cashmere fiber diameters and yields for cashmere goat (9). Despite some reports demonstrating that specific amino acids, including lysine and histidine, play important roles in milk production and milk protein synthesis in goats (10, 11), the types of limiting amino acids in goats are not currently understood fully.

Post-transcriptional modifications (PTMs) refer to a variety of vital biological processes in cells that can alter the physicochemical properties of a protein to alter its functions, and phosphorylation represents one of the most extensive and well-studied PTMs, occurring ubiquitously throughout cellular regulation systems and impacting approximately one-third of all eukaryotic proteins (12, 13). Phosphorylation typically occurs at specific serine, threonine, and tyrosine residues of eukaryotic proteins, which occupy over 98% of phosphorylation sites, through the actions of site-specific protein kinases (12, 14, 15). Phosphorylated proteins play roles in almost all cellular biological processes, including cell growth, differentiation, metabolism, migration, autophagy, apoptosis, and disease progression (16–19). Phosphorylation is also commonly involved in many signal transduction processes, such as the mitogen-activated protein kinase (MAPK) signaling pathway. Extracellular signals, such as the interactions between growth factors and cell-surface receptors, are transmitted along the Ras-MAPKKK-MAPKK-MAPK axis through cascading phosphorylation events to regulate cell survival, proliferation, differentiation, and apoptosis (20–25).

Studies of the amino acid response (AAR) signaling pathway have revealed that amino acid deficiencies can contribute to the accumulation of intracellular uncharged tRNA, which can induce the phosphorylation of eukaryotic initiation factor (eIF)2α at ser51 by activated general control non-derepressible 2 (GCN2) (26–29). In addition, amino acids have been shown to participate in the mammalian/mechanistic target of rapamycin complex 1 (mTORC1) signaling pathway through a phosphorylation cascade. Amino acid signals can activate mTORC1, which then phosphorylates downstream effectors, including eIF4E-binding protein 1 (4EBP1), Unc-51-like autophagy activating kinase 1 (ULK1), and ribosomal protein S6 kinase beta-1 (S6K1) to regulate cell metabolism and autophagy (19, 30). However, only two types of amino acid signals, leucine and arginine, are currently known to activate mTORC1 to induce the intracellular phosphorylation cascade along this signaling pathway (31, 32). Whereas, the mechanisms through which other amino acid signals are detected have yet to be clearly elucidated.

Arbas white Cashmere goats (also known as Inner Mongolia Cashmere goats) represent an outstanding dual-purpose breed of goat, providing both cashmere and meat, and amino acid nutritional characteristics can significantly influence the production of high-quality cashmere and meat. In the present study, phosphorylation-based proteomics was used to analyze the changes in the phosphorylation levels of proteins and the activation of dynamic intracellular networks in response to amino acid nutrition in Cashmere goat fetal fibroblasts. Pivotal and previously unreported phosphosites and phosphorylated proteins were identified, and the intracellular interaction networks that were activated by amino acid signals were predicted. Three membrane proteins, the zinc transporter SLC39A7, the sodium-dependent neutral amino acid transporters SLC1A5 and SLC38A7, three translation initiation factors (eIF5B, eIF4G, and eIF3C), and heat-shock protein (HSP)90A, which have rarely been reported in association with amino acid signaling mechanisms, were added to the currently known signaling pathways, including the GCN2 pathway and the mTORC1 pathway, to update the model of amino acid signal-inducing pathways in Cashmere goat fetal fibroblasts. The results of this study provided new insights into the precise mechanisms through which amino acid nutrition are sensed by goat cells.

## Materials and Methods

### Ethics Statement

All experimental procedures involving animals were conducted according to the guidelines for the care and use of experimental animals that have been established by the Inner Mongolia University Animal Care and Use Committee.

### Cell Culture

Goat fetal fibroblasts (GFbs) were obtained from Arbas White Cashmere goats. The procedures used to isolate and characterize of GFbs were performed as previously described (33). GFb cell cultures were maintained as previously described (34). Briefly, GFb cells were cultured as monolayer cultures in Dulbecco's modified Eagle medium (DMEM)/F12 (GE Healthcare Life Sciences, Logan, Utah, U.S.A.) supplemented with 10% fetal bovine serum (Sigma-Aldrich, Saint Louis, Missouri, U.S.A.) and 1% penicillin-streptomycin (100×) (TransGen Biotech, Beijing, China). Cell lines were maintained and incubated at 37°C in a humidified incubator with 5% CO_2_.

### Reagents and Antibodies

Leucine powder (Sigma-Aldrich, Saint Louis, Missouri, U.S.A.) was dissolved in amino acid-free DMEM/F12 medium (GE Healthcare Life Sciences, Logan, Utah, U.S.A.), at a concentration of 100 mM. The following primary antibodies were used: anti-β-actin (Sigma-Aldrich, Saint Louis, Missouri, U.S.A.), anti-ULK1 (Cell Signaling Technology, Danvers, Massachusetts, U.S.A.), anti-pho-ULK1 Ser757 (Cell Signaling Technology, Danvers, Massachusetts, U.S.A.), anti-mTOR (Abcam, Cambridge, UK), anti-pho-mTOR Ser2448 (Abcam, Cambridge, UK), anti-LC3A/B (Cell Signaling Technology, Danvers, Massachusetts, U.S.A.), anti-pho-p44/42 MAPK Thr202/Tyr204 (Cell Signaling Technology, Danvers, Massachusetts, U.S.A.), anti-MAPK (Cell Signaling Technology, Danvers, Massachusetts, U.S.A.), anti-mouse IgG-HRP, and anti-rabbit IgG-HRP (GE Healthcare Life Sciences, Logan, Utah, U.S.A.).

### Experimental Procedures for Cell Processing

GFb cells were grown in six-well plates to 80–90% confluence. For amino acid starvation experiment used to induce autophagy, the non-treated group (NT group) was maintained in DMEM/F12 medium supplemented with 10% fetal bovine serum for 16 h, the serum starvation group (SS group) was maintained in serum-free DMEM/F12 medium for 16 h, and the S1h, S2h, and S4h groups were maintained in serum-free DMEM/F12 medium for 16 h followed by incubation with amino acid-free DMEM/F12 for 1, 2, and 4 h, respectively. For amino acid starvation experiment used to attenuate mTORC1/ULK1 pathway, the non-treated group (NT group) and serum starvation group (SS group) were treated as described above, and AAS groups were maintained in serum-free DMEM/F12 medium for 16 h followed by incubation with amino acid-free DMEM/F12 for 1 h. To examine the activation of the mTORC1/ULK1 pathway in response to leucine, the amino acid starvation group (AAS group) was maintained in serum-free DMEM/F12 medium for 16 h, followed by incubation in amino acid-free DMEM/F12 for 1 h whereas Leu200 and Leu450 groups were maintained in serum-free DMEM/F12 medium for 16 h, incubated in amino acid-free DMEM/F12 for 1 h, and then incubated in amino acid-free DMEM/F12 supplemented with 200 and 450 μM leucine, respectively, for 1 h to initiate leucine restimulation. To examine the response of the mTORC1/ULK1 pathway to all amino acids and validate the specific phosphosites and phosphoproteins associated with amino acid signaling pathways, an AAS group was generated as described above, and amino acid restimulation conditions were applied as described for leucine restimulation, in which cells were serum-starved, amino acid-starved, and then incubated for 1 h in DMEM/F12 with 250 μM glycine, 50 μM alanine, 700 μM arginine, 50 μM asparagine, 50 μM aspartic acid, 100 μM cysteine, 100 μM cystine, 50 μM glutamic acid, 2,500 μM glutamine, 150 μM histidine, 416 μM isoleucine, 451 μM leucine, 499 μM lysine, 116 μM methionine, 215 μM phenylalanine, 150 μM proline, 250 μM serine, 449 μM threonine, 44 μM tryptophan, 214 μM tyrosine, and 452 μM valine to generate an all amino acid restimulation group (AllS group). At the end of each experiment, the cells were lysed in cell lysis buffer (Beyotime, Shanghai, China), composed of 50 mM Tris (pH 7.4), 150 mM NaCl, 1% NP-40, 0.5% sodium deoxycholate, 0.1% SDS, 1% phenylmethylsulfonyl fluoride (PMSF, Beyotime, Shanghai, China), 1% phosphatase inhibitor cocktail 2 (Sigma-Aldrich, Saint Louis, Missouri, U.S.A.), and 1% phosphatase inhibitor cocktail 3 (Sigma-Aldrich, Saint Louis, Missouri, U.S.A.), for 10 min at 4°C. The cell lysates were collected and centrifugated at 12,000 rpm at 4°C in a microcentrifuge for 10 min, and then the supernatant was collected and stored at −80°C for western blot analysis.

To obtain protein samples for the phosphoproteomics analysis, GFb cells were grown in 10 cm dishes to 80–90% confluence. Cells were divided into five groups, including NT, SS, AAS, LeuS, and AllS groups, which were treated as described above and leucine restimulation concentration is 450 μM in LeuS group. All groups were treated simultaneously. At the end of the treatment protocol, the cells were lysed using cell lysis buffer supplemented with 1% PMSF, 1% phosphatase inhibitor cocktail 2, and 1% phosphatase inhibitor cocktail 3, as described above. All protein samples were frozen at −80°C, immediately after centrifugation. Three replicates were processed for each group, and three biologically duplicated protein samples were mixed to perform liquid chromatography-tandem mass spectrometry (LC-MS/MS) analysis.

### Western Blot Analysis

Western blot was used to detect the expression levels of the indicated proteins and phosphorylated proteins, as previously described (35). Briefly, the supernatants were collected and boiled at 95°C with shaking for 10 min. Proteins were separated by sodium dodecyl sulfate-polyacrylamide gel electrophoresis (SDS-PAGE), transferred to a polyvinylidene difluoride (PVDF) membrane (Bio-Rad, Hercules, California, U.S.A.), and blocked with 5% skimmed milk powder (Becton, Dickinson and Company, Sparks, Maryland, U.S.A.) in Tris-buffered saline containing Tween 20 (TBST) for 1 h at 37°C, before incubation with primary antibodies at 4°C overnight. PVDF membranes were then incubated with horseradish peroxidase (HRP)-conjugated secondary antibodies at room temperature for 1 h, and then visualized with enhanced chemiluminescence (ECL) reagent (SageCreation, Beijing, China). Protein bands were detected using the Minochemi™ Chemiluminescence Imaging System and SagaCapture software (SageCreation, Beijing, China).

### Immunofluorescence Staining

GFb cells were cultured on coverslips in a 6-well plate at 50–60% confluence. Cells were treated as described above to obtain NT, SS, and AAS groups. Cells were fixed for 15 min with 4% paraformaldehyde and then stained for 30 min with monodansylcadaverin (MDC) (KeyGEN BioTECH, Nanjing, China) and 3 min with 4′,6-diamidino-2-phenylindole (DAPI, Beyotime, Shanghai, China) at room temperature. Cells were washed three times with phosphate-buffered saline (PBS) at the end of each step. Finally, slides were sealed with glycerinum and observed under a laser-scanning confocal microscope (NIKON A1R, Nikon Corp., Tokyo, Japan).

### Phosphoproteomics Profiling

Protein samples were subjected to reductive alkylation treatment with iodoacetamide to break disulfide bonds. Next, trypsin was used to digest the proteins into short peptides. Then, TiO_2_ was used to enrich phosphopeptides. Phosphopeptides were separated on a 50 cm reverse phase column (in-house packed with Dr. Maisch GmbH reversed-phase beads 1.9 μm Reprosil-Pur 120 C18-AQ) over 270 min. Eluting phosphopeptides were analyzed by the quadrupole orbitrap mass spectrometer (Q Exactive HF, Thermo Fisher Scientific). The spectra of first-grade MS and second-grade MS were obtained as following parameter: resolution = 70,000 and 17,500, automatic gain control target = 3e6 and 1e5, maximum injection time = 40 and 40 ms. High-energy collisional dissociation (HCD) were performed by second-grade MS, and the normalized collision energy is 30 eV. The raw data were analyzed using ProteinPilot software, based on the results of protein database searches to identify proteins. Based on quality control measures, we were able to obtain reliable peptides and proteins. The phosphosites were screened out according to the molecular weights of the phosphoric acid group.

### The Screening Process Used to Identify Phosphosites and Phosphoproteins

The phosphosites shared between the SS group and the AAS group, between the AAS group and the LeuS group, and between the AAS group and the AllS group were analyzed and screened. The phosphorylation abundance of phosphosites in the AAS group was compared with that in the SS group to generate an ratio in the AAS group vs. SS group, for which a value > 1 at any phosphosite indicated that amino acid starvation caused the upregulation of phosphorylation at that site, whereas a ratio of <1 indicated the down-regulation of the phosphorylation level at this site. Similar comparisons were performed for the AAS group vs. LeuS group and the AAS group vs. AllS group. Ratios > 2 or <0.5, which represented |log_2_(FC)| > 1, were used to filter phosphosites that were upregulated in the AAS group vs. SS group comparison and downregulated in the LeuS group vs. AAS group comparison, which were added to the dataset Frame 1 as phosphosites whose phosphorylation levels are increased by amino acid starvation and decreased by leucine restimulation, indicating that leucine can negatively regulate the phosphorylation of these sites. A similar approach was used to identify those phosphosites at which phosphorylation was positively regulated by leucine (Frame 2), negatively regulated by all amino acids (Frame 3), and positively regulated by all amino acids (Frame 4).

### Bioinformatics Analysis

Goat genome annotation for proteins was obtained from the Ensembl database (Capra_hircus. ARS1). Peptide scoring and protein identification were measured using ProteinPilot software, and the relative quantification of phosphopeptides was performed using PeakView software. Venn diagrams were generated using the R software Venndiagram package. Heatmaps were generated using the R software Heatmap package. Gene ontology (GO) and Kyoto Encyclopedia of Genes and Genomes (KEGG) functional annotations were performed using the R software ClusterProfiler package (36). Interaction network analysis was performed using the String database (https://string-db.org/) and Cytoscape software. MoMo software from MEME (http://meme-suite.org/tools/momo) was used to identify phosphosite motifs (37).

### Statistical Analyses

Statistical analyses were conducted using SPSS PASW Statistics for Windows, v18.0 (SPSS Inc.: Chicago, IL, U.S.A.). Western blot results were quantified using Gel-Pro Analyzer 4.0 (Media Cybernetics, U.S.A.), and the quantitative values were compared using a *t*-test. Data were expressed as the mean ± standard deviation (SD). Significance was accepted at *p* < 0.05. All results represent the average of at least three independent experiments unless stated otherwise.

### Data Availability

The mass spectrometry data have been deposited in the ProteomeXchange database (http://www.proteomexchange.org/) and the accession number is PXD025488.

## Results

### Amino Acid Starvation Induces Autophagy in Goat Fetal Fibroblasts

**Figure 1:**
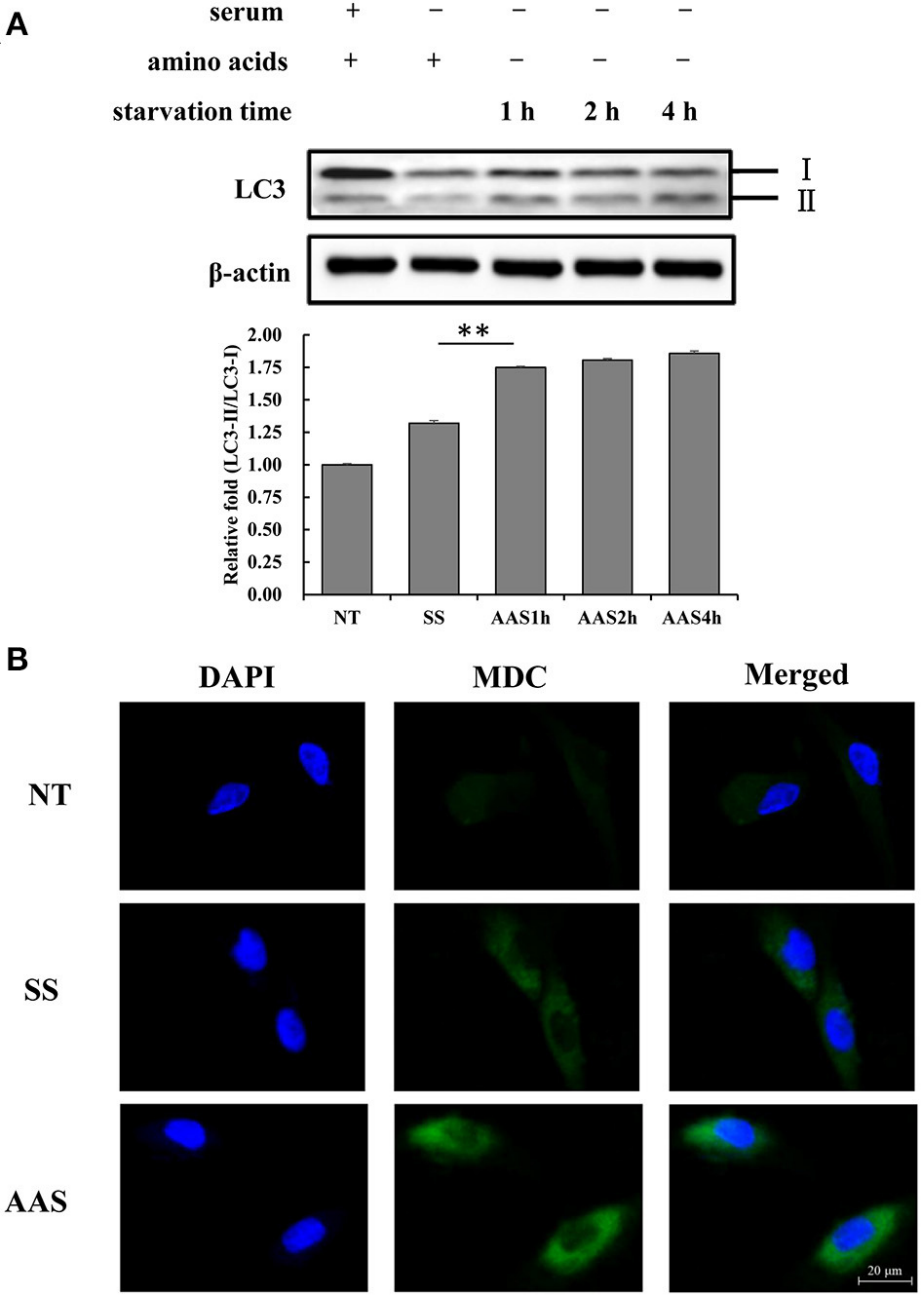
Amino acid starvation induces cell autophagy in goat fetal fibroblasts. **(A)** The proteins were extracted from cells in non-treated condition (NT group), serum starvation medium for 16 h (SS group), amino acid starvation medium for 1 h after serum starvation (AAS1h group), amino acid starvation medium for 2 h after serum starvation (AAS2h group), and amino acid starvation medium for 4 h after serum starvation (AAS4h group). Then, the expression of LC3 protein was analyzed by immunoblotting. Amino acid starvation enhanced the LC3-II/I ratio. **(B)** GFb cells were in non-treated condition (NT group), serum starvation medium for 16 h (SS group), amino acid starvation medium for 1 h after serum starvation (AAS group), followed by immunofluorescence staining with MDC and DAPI. The cells were observed by a laser-scanning confocal microscope. Amino acid starvation increased the formation of autophagic vacuoles. All bands were quantified using Gel-Pro Analyzer 4.0 (^*^p < 0.05, ^**^p < 0.01, *n* = 3 experiments).

To examine the extent of autophagy induced by amino acid deficiency, GFb cells were subjected to amino acid starvation conditions for 1, 2, and 4 h. The results showed that the LC3-II/LC3-I ratio gradually increased with increasing amino acid starvation treatment times (Figure 1A), which indicated the conversion of LC3 from the cytoplasmic form to the membrane-localized form, which is an indicator of increased autophagy. The numbers of autophagic vesicles in GFb cells were compared among groups, and more autophagic vesicles were observed in the AAS group than in the NT and SS groups (Figure 1B), which suggested that amino acid deficiency can induce autophagy in GFb cells.

### mTORC1/ULK1 Pathway Responses to Amino Acid Nutrition in Goat Fetal Fibroblasts

**Figure 2:**
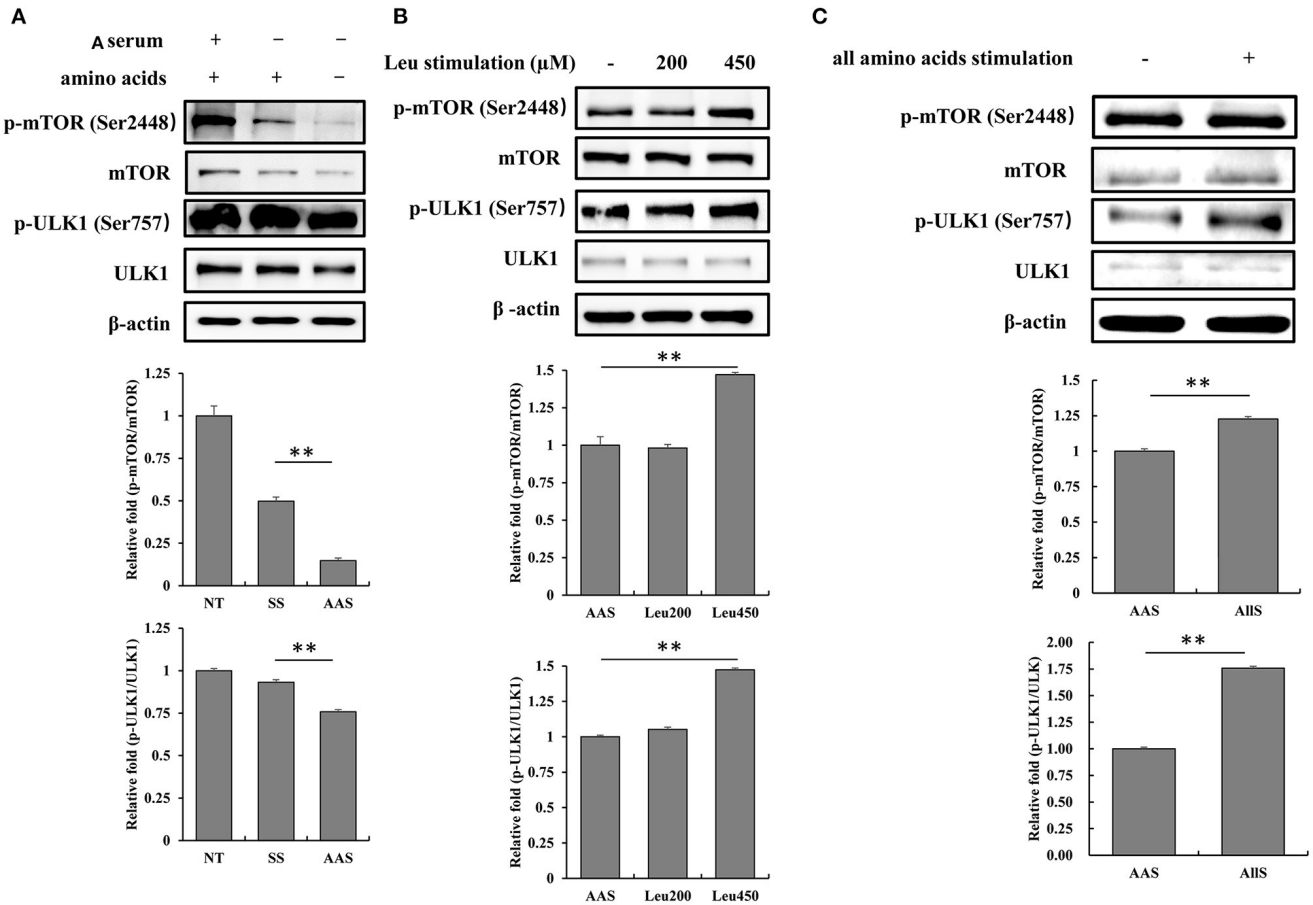
Amino acid starvation and restimulation with amino acids influenced mTORC1 activity in goat fetal fibroblasts. **(A)** The proteins were extracted from cells in non-treated condition (NT group), serum starvation medium for 16 h (SS group), and amino acid starvation medium for 1 h after serum starvation (AAS group). Then, the expression and phosphorylation levels of mTOR and ULK1 proteins were analyzed by immunoblotting. mTORC1/ULK1 signaling was attenuated by amino acid starvation. **(B)** GFb cells were subjected to amino acid starvation for 1 h, after serum starvation for 16 h, and then restimulated with leucine at concentrations of 200 and 450 μM. The cells were lysed, and the expression and phosphorylation levels of mTOR and ULK1 proteins were analyzed by immunoblotting. The restimulation with leucine improved mTORC1/ULK1 signaling. **(C)** GFb cells were subjected to amino acid starvation for 1 h, after serum starvation for 16 h, and then restimulation with all amino acids. The cells were lysed, and the expression and phosphorylation levels of mTOR and ULK1 proteins were analyzed by immunoblotting. The restimulation with all amino acids enhanced mTORC1/ULK1 signaling. All bands were quantified using Gel-Pro Analyzer 4.0 (^*^p < 0.05, ^**^p < 0.01, *n* = 3 experiments).

To evaluate the influence of amino acid deficiency on the mTORC1/ULK1 signaling pathway, GFb cells were subjected to amino acid starvation for 1 h, and the phosphorylation levels of mTOR and ULK1 were detected by western blot analysis. The results showed that the phosphorylation levels of mTOR and ULK1 weakened after amino acid starvation, compared with the levels observed in the NT and SS groups (Figure 2A), which indicated the inhibition of the mTORC1/ULK1 signaling pathway under amino acid-deficient conditions.

To confirm the mTORC1/ULK1 signaling pathway response to amino acid nutrition, GFb cells were restimulated after amino acid starvation with either leucine, at concentrations of 200 and 450 μM, or all amino acids incluing 21 kinds of amino acids, for 1 h. The results showed that mTOR and ULK1 phosphorylation levels were significantly enhanced by restimulation with both 450 μM leucine alone and all amino acids (Figures 2B,C), which indicated that mTORC1/ULK1 signaling was activated by amino acid signals.

### Identification of Phosphosites and Phosphoproteins in Goat Fetal Fibroblasts

**Figure 3:**
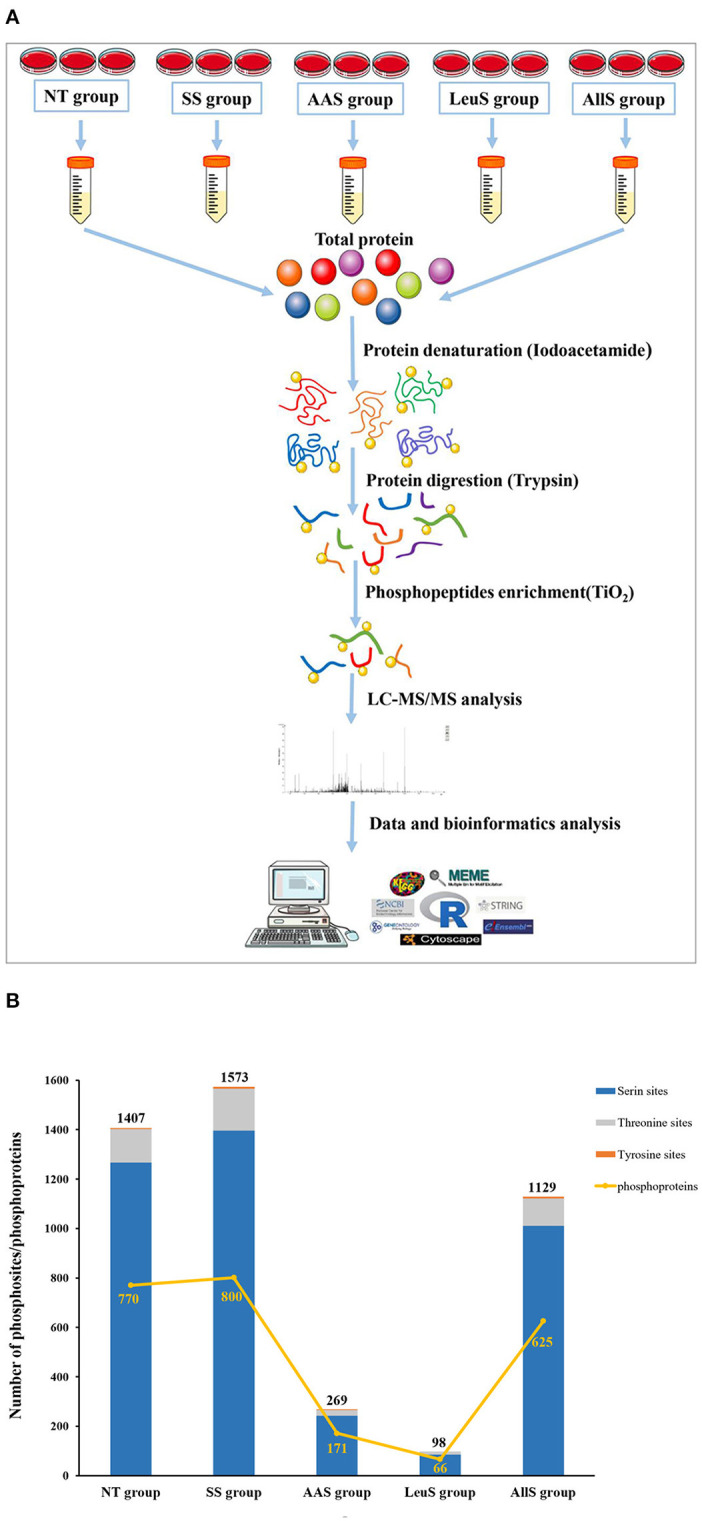
Procedures and basic identification information for the phosphoproteomics analysis. **(A)** Abridged general view of the phosphoproteomics analysis. The proteins were extracted from cells in non-treated condition (NT group), serum starvation medium for 16 h (SS group), amino acid starvation medium for 1 h after serum starvation (AAS group), 450 μM leucine restimulation medium for 1 h after serum and amino acid starvation (LeuS), and all amino acid restimulation medium for 1 h after serum and amino acid starvation (AllS group). Then, proteins were subjected to reductive alkylation treatment using iodoacetamide, followed by digestion into short peptides using trypsin. Subsequently, phosphatides were enriched using TiO_2_ and detected by LC-MS/MS. Proteins were identified by ProteinPilot software, and bioinformatics analyses were performed using R software, Cytoscape software, and MoMo software. **(B)** The numbers of phosphosites in the NT, SS, AAS, LeuS, and AllS groups were 1,407, 1,573, 269, 98, and 1,129, respectively. The numbers of phosphoproteins identified in the NT, SS, AAS, LeuS, and AllS groups were 770, 800, 171, 66, and 625, respectively.

To determine the specific phosphosites and phosphoproteins found in the NT, SS, AAS, LeuS, and AllS groups, GFb cells from all five groups were processed as described in Materials and Methods. The protein samples were subjected to reductive alkylation treatment with iodoacetamide to break disulfide bonds, followed by digestion into short peptides by trypsin. Finally, phosphopeptides were enriched by TiO_2_ treatment. LC-MS/MS-based quantitative phosphoproteomics was used to analyze the isolated phosphopeptides. The experimental procedure is displayed in Figure 3A. Peptide scoring and protein identification were performed using ProteinPilot software, and the relative quantification of the phosphorylated peptides was performed using PeakView software. From the NT, SS, AAS, LeuS, and AllS groups, we obtained 2,102, 2,657, 337, 127, and 1,727 phosphopeptides, respectively, associated with 881, 925, 184, 72, and 702 phosphoproteins (Table 1). We normalized the quantitative data and removed redundant and unreliable data. Then, all phosphopeptides were compared against the database, and phosphopeptides containing the same phosphosites for each protein were merged. Ultimately, 1,407, 1,573, 269, 98, and 1,129 unique phosphosites were identified for the quantitative analysis, and the proportions represent that phosphorylated serine were 90.05, 88.75, 89.96, 87.76, and 89.55%, and phosphorylated threonine were 9.59, 10.81, 8.92, 12.24, and 9.83% for the NT, SS, AAS, LeuS, and AllS groups, respectively (Figure 3B and Table 2).

**Table 1:** The composition and number of identified petides and proteins.

**Groups**	**Phosphopeptides**	**Total peptides**	**Phosphopeptides%**	**Phosphoproteins**	**Total proteins**	**Phosphoproteins%**
NT group	2,102	3,631	57.89%	881	1,230	71.63%
SS group	2,657	5,806	45.76%	925	1,461	63.31%
AAS group	337	528	63.83%	184	266	69.17%
LeuS group	127	202	62.87%	72	112	64.29%
AllS group	1,727	2,958	58.38%	702	989	70.98%

**Table 2:** The compositon and number of phosphosites and phosphoproteins.

	**Serine sites**		**Threonine sites**		**Tyrosine sites**		**Phosphosites**	**Phosphoproteins**
NT group	1,267	90.05%	135	9.59%	5	0.36%	1,407	770
SS group	1,396	88.75%	170	10.81%	7	0.45%	1,573	800
AAS group	242	89.96%	24	8.92%	3	1.12%	269	171
LeuS group	86	87.76%	12	12.24%	0	0.00%	98	66
AllS group	1,011	89.55%	111	9.83%	7	0.62%	1,129	625

### Screening for Differentially Abundant Phosphosites

**Figure 4:**
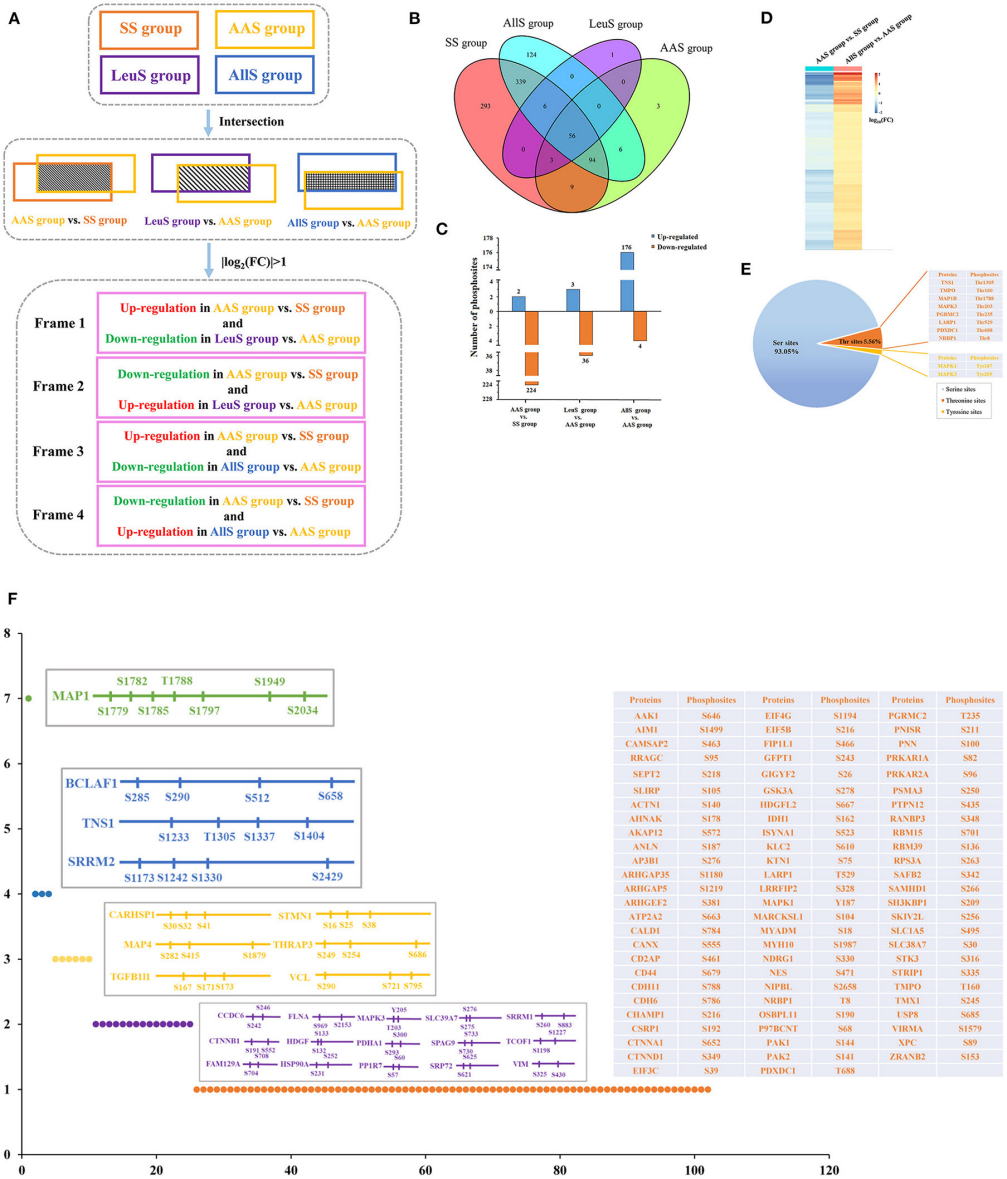
Screening of phosphosites and phosphoproteins associated with amino acid signaling. **(A)** Concise schematic diagram of the screening process. The intersecting phosphosites between the SS and AAS groups, between the AAS and LeuS groups, and between the AAS and AllS groups were examined. Subsequently, |log_2_(FC)| > 1 was used as a filter, and changes in phosphorylation levels were compared between the AAS group vs. SS group and LeuS group vs. AAS group, as well as AAS group vs. SS group and AllS group vs. AAS groups, to obtain phosphosites in Frames 1–4. **(B)** The Venn diagram indicates that the number of proteins shared between the SS and AAS groups is 162, between the AAS and LeuS groups is 59, and between the AAS and AllS groups is 156. **(C)** The numbers of phosphosites that were upregulated in the comparisons AAS group vs. SS group, LeuS group vs. AAS group, and AllS group vs. AAS group were 2, 3, and 176, respectively. The numbers of phosphosites that were downregulated in the comparisons AAS group vs. SS group, LeuS group vs. AAS group, and AllS group vs. AAS group were 224, 36, and 4, respectively. **(D)** Heatmap, indicating the fold-changes in the phosphorylation abundance of phosphosites positively associated with the amino acid signals. The heatmap for the comparison AAS group vs. SS group shows declines in the phosphorylation levels after amino acid starvation, whereas the heatmap for the comparison AllS group vs. AAS group shows the increasing phosphorylation levels after restimulation with all amino acids. Blue represents a decrease in phosphorylation, whereas orange represents an increase in phosphorylation. Darker colors indicate greater fold changes. **(E)** The composition of phosphosites was positively associated with the amino acid signals. The proportions of serine, threonine, and tyrosine sites among all phosphosites were 93.05, 5.56, and 1.39%, respectively. **(F)** The distribution of phosphosites on proteins was positively associated with the amino acid signals. Most of the phosphosites were evenly distributed across different proteins. Only 15, 6, 3, and 1 protein featured 2, 3, 4, and 7 phosphosites, respectively.

As demonstrated showed above, the phosphorylation abundance within the proteome was extremely different between the SS, AAS, LeuS, and AllS groups. Therefore, it was necessary to analyze those proteins whose phosphorylation abundance changed both after amino acid starvation and after restimulation by leucine or all amino acids. The data processing procedures are illustrated in Figure 4A. In brief, the phosphorylation levels of those sites included in Frame 1 were negatively regulated by the leucine signal, whereas those sites in Frame 2 were positively regulated by the leucine signal. Similarly, the phosphorylation levels of those sites in Frame 3 were negatively regulated by all amino acid signals, whereas those sites in Frame 4 were positively regulated by all amino acid signals.

All phosphoproteins identified in the SS, AAS, LeuS, and AllS groups were counted using the R software Venndiagram package. In total, there were 162, 59, and 156 proteins in the intersection between SS and AAS groups, AAS and LeuS groups, as well as AAS and AllS groups, respectively (Figure 4B), showing that these proteins may be correlated with amino acid starvation signal, leucine stimulation signal and all amino acids stimulation signal. Then relative abundance of phosphorylation of sites on proteins mentioned above was calculated and counted. When comparing the AAS group with the SS group, 2 sites were associated with upregulated phosphorylation, and 224 sites were associated with downregulated phosphorylation, which showed that amino acid starvation signal significantly decreased the phosphorylation level of proteins. When comparing the LeuS group with the AAS group, 3 sites were associated with upregulated phosphorylation, and 36 sites were associated with downregulated phosphorylation. When the AllS group was compared with the AAS group, 176 sites were associated with upregulated phosphorylation, and 4 sites were associated with downregulated phosphorylation, which indicated that all amino acids appreciably enhanced the phosphorylation level of proteins (Figure 4C). These results suggested that amino acid signals positively regulated the phosphorylation status on the majority of the identified phosphoproteins.

Using the screening process described above, 2 sites for which the phosphorylation levels were negatively regulated by leucine were identified in Frame 1, and 2 sites for which phosphorylation levels were positively regulated by leucine were identified in Frame 2 (Table 3), showing that the phosphorylation of serine 722 on erythrocyte membrane protein band 4.1 like 2 (EPB41L2), threonine 220 on serine/arginine repetitive matrix 1 (SRRM1), serine 252 on HSP90A and serine 784 on caldesmon 1 (CALD1) were closely related to leucine signal. Frame 3 featured an empty set, which indicated that no sites were identified for which the phosphorylation levels were negatively regulated by all amino acids, whereas 144 sites were identified in Frame 4, for which the phosphorylation levels were positively regulated by all amino acids. A total of 17 phosphosites with |log_10_(FC)| > 1, associated with 12 proteins, were identified in Frame 4, as shown in Table 4, which indicated that the phosphorylation of serine 646 on AP2 associated kinase 1 (AAK1), serine 1499 on absent in melanoma 1 (AIM1), serine 1779 and 1782 on microtubule associated protein 1 (MAP1), serine 512 on Bcl-2-associated transcription factor 1 (BCLAF1), serine 242 and 246 on coiled-coil domain containing 6 (CCDC6), serine 969 on filamin A (FLNA), serine 231 and 252 on HSP90A, tyrosine 187 on MAPK1, serine 1987 on myosin heavy chain 10 (MYH10), threonine 688 on pyridoxal dependent decarboxylase domain containing 1 (PDXDC1), serine 25 on stathmin 1 (STMN1), as well as serine 167, 171, and 173 on transforming growth factor beta-1-induced transcript 1 (TGFB1I1) was significantly correlated to all amino acids. Meanwhile, the fold change of phosphorylation abundance was calculated for all identified sites in Frame 4, as demonstrated in Figure 4D and Supplementary Table 1. In Frame 4, 134 serines were identified on 95 proteins, 8 threonines were identified on 8 proteins, and 2 tyrosines were identified on 2 proteins (Figure 4E). The distribution of phosphosites on the proteins identified in Frame 4 were analyzed, and the results were as follows: microtubule-associated protein 1 (MAP1) featured 7 phosphorylation sites, including 6 serines and 1 threonine; BCLAF1, tensin-1 (TNS1), and serine/arginine repetitive matrix 2 (SRRM2) each featured 4 phosphorylation sites; calcium-regulated heat-stable protein 1 (CARHSP1), microtubule-associated protein 4 (MAP4), STMN1, TGFB1I1, thyroid hormone receptor-associated protein 3 (THRAP3), and vinculin (VCL) each featured 3 serine phosphorylation sites; and the remaining 15 and 77 proteins each featured two and one phosphorylation site, respectively (Figure 4F). These data indicated that proteins associated with larger fold-changes in phosphorylation levels and multiple phosphorylation sites might represent key proteins in the intracellular signaling pathways induced by all amino acid signals.

**Table 3:** Relative quantitative data of phosphosites responding to leucine signal.

**Data set**	**Protein names**	**Protein ID**	**Phosphosites**	**Fold change** **(AAS group vs. SS group)**	**Fold change** **(LeuS group vs. AAS group)**
Frame 1	EPB41L2	chx:102190693	Ser722	2.35	0.06
	SRRM1	chx:102175909	Thr220	2.26	0.11
Frame 2	HSP90A	chx:100860851	Ser252	0.01	6.11
	CALD1	chx:102188560	Ser784	0.22	2.33

**Table 4:** Relative quantitative data of phosphosites (|log_10_(FC)| > 1) responding to all amino acids signal.

**Protein names**	**Protein ID**	**Phosphosites**	**Fold change** **(AAS group vs. SS group)**	**Fold change** **(AllS group vs. AAS group)**
AAK1	chx:102187599	Ser646	0.02	41.89
AIM1	chx:102180395	Ser1499	0.06	14.38
MAP1	chx:102176074	Ser1782	0.02	33.16
MAP1	chx:102176074	Ser1779	0.04	16.53
BCLAF1	chx:102180497	Ser512	0.02	26.17
CCDC6	chx:102172968	Ser246	0.06	24.07
CCDC6	chx:102172968	Ser242	0.06	14.31
FLNA	chx:102176601	Ser969	0.08	13.53
HSP90A	chx:100860851	Ser252	0.01	23.49
HSP90A	chx:100860851	Ser231	0.08	12.89
MAPK1	chx:102186040	Tyr187	0.06	12.80
MYH10	chx:102177371	Ser1987	0.05	16.61
PDXDC1	chx:102176932	Thr688	0.02	44.23
STMN1	chx:102173139	Ser25	0.02	113.30
TGFB1I1	chx:100861032	Ser173	0.04	147.26
TGFB1I1	chx:100861032	Ser167	0.02	11.80
TGFB1I1	chx:100861032	Ser171	0.02	10.71

### GO and KEGG Analysis of Proteins Based on Changes in Phosphorylation Levels in Response to Amino Acid Signals

**Figure 5:**
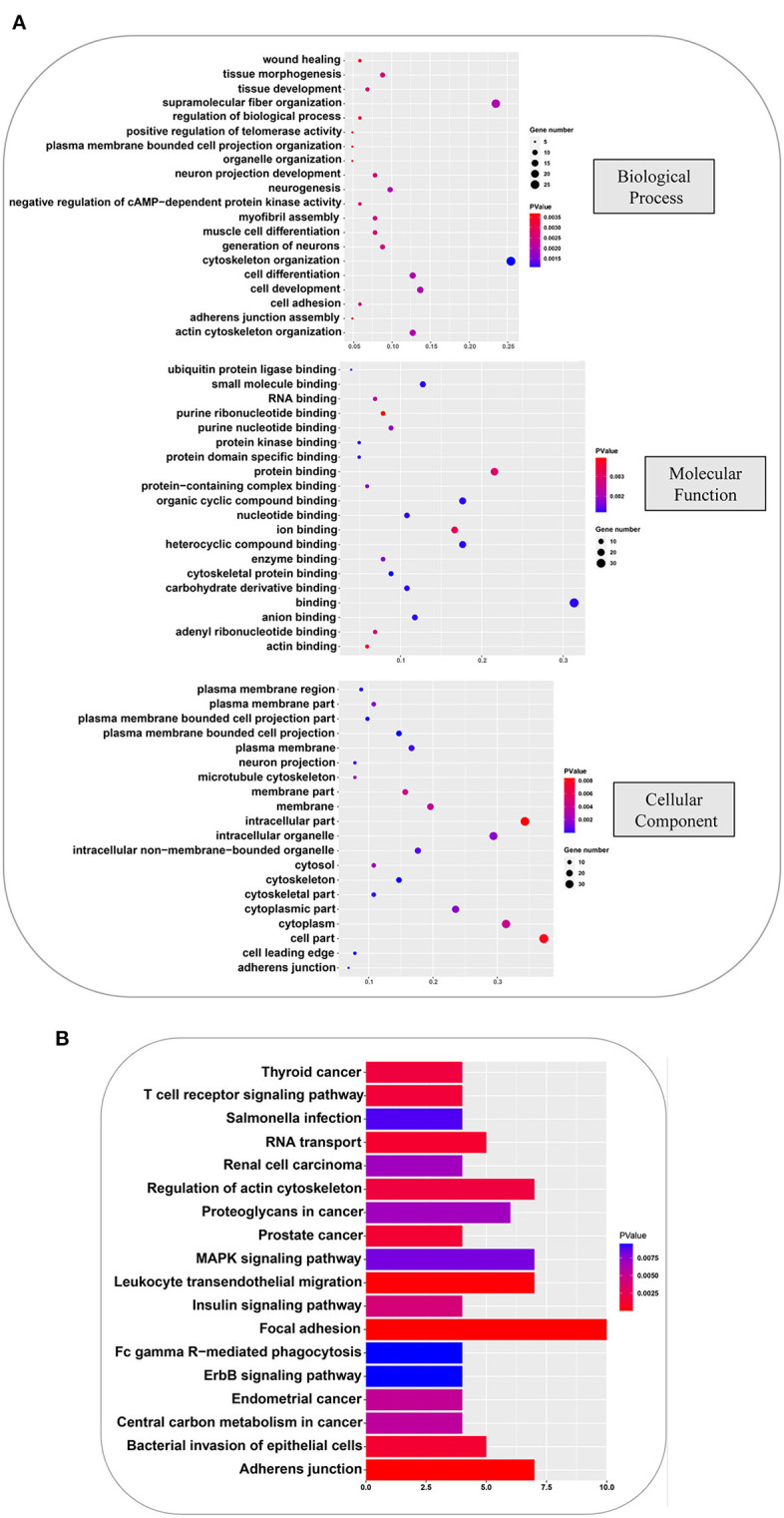
GO and KEGG analyses, based on the phosphorylated proteins that were positively associated with the amino acid signals. **(A)** GO analysis of phosphorylated proteins positively associated with all amino acids, including: biological processes, such as cytoskeleton organization, supramolecular fiber organization, cell development, cell differentiation, and actin cytoskeleton organization; molecular function, such as binding, protein binding, organic cyclic compound binding, heterocyclic compound binding, and ion binding; and cellular component, such as cell part, intracellular part, cytoplasm, intracellular organelle, and cytoplasmic part. **(B)** KEGG pathway analysis of phosphorylated proteins that were positively associated with all amino acids, indicating that focal adhesion, MAPK signaling pathway, adherent junction, leukocyte transendothelial migration, and regulation of actin cytoskeleton were principal terms.

To further understand the physiological functions and significance of those proteins whose phosphorylation was positively regulated by all amino acids, GO and KEGG functional annotation analyses were performed using R software ClusterProfiler package. Proteins with significantly different phosphorylation levels were enriched in 133 GO terms and 18 KEGG pathways. The GO analysis showed that these proteins were primarily enriched in biological processes, including cytoskeleton organization, supramolecular fiber organization, cell development, cell differentiation, and actin cytoskeleton organization; in molecular function, including binding, protein binding, organic cyclic compound binding, heterocyclic compound binding, and ion binding; and cellular components, including cell part, intracellular part, cytoplasm, intracellular organelle, and cytoplasmic part. The top 20 GO terms are shown in Figure 5A. Focal adhesion, MAPK signaling pathway, adherent junction, leukocyte transendothelial migration, and regulation of actin cytoskeleton were the principal terms identified by the KEGG analysis (Figure 5B). These analyses showed that the phosphorylation cascaded induced by amino acid signals participates in many physiological and biochemical process in GFb cells.

### Interaction Network Analysis of Identified Phosphorylated Proteins Correlated With Amino Acid Signals

**Figure 6:**
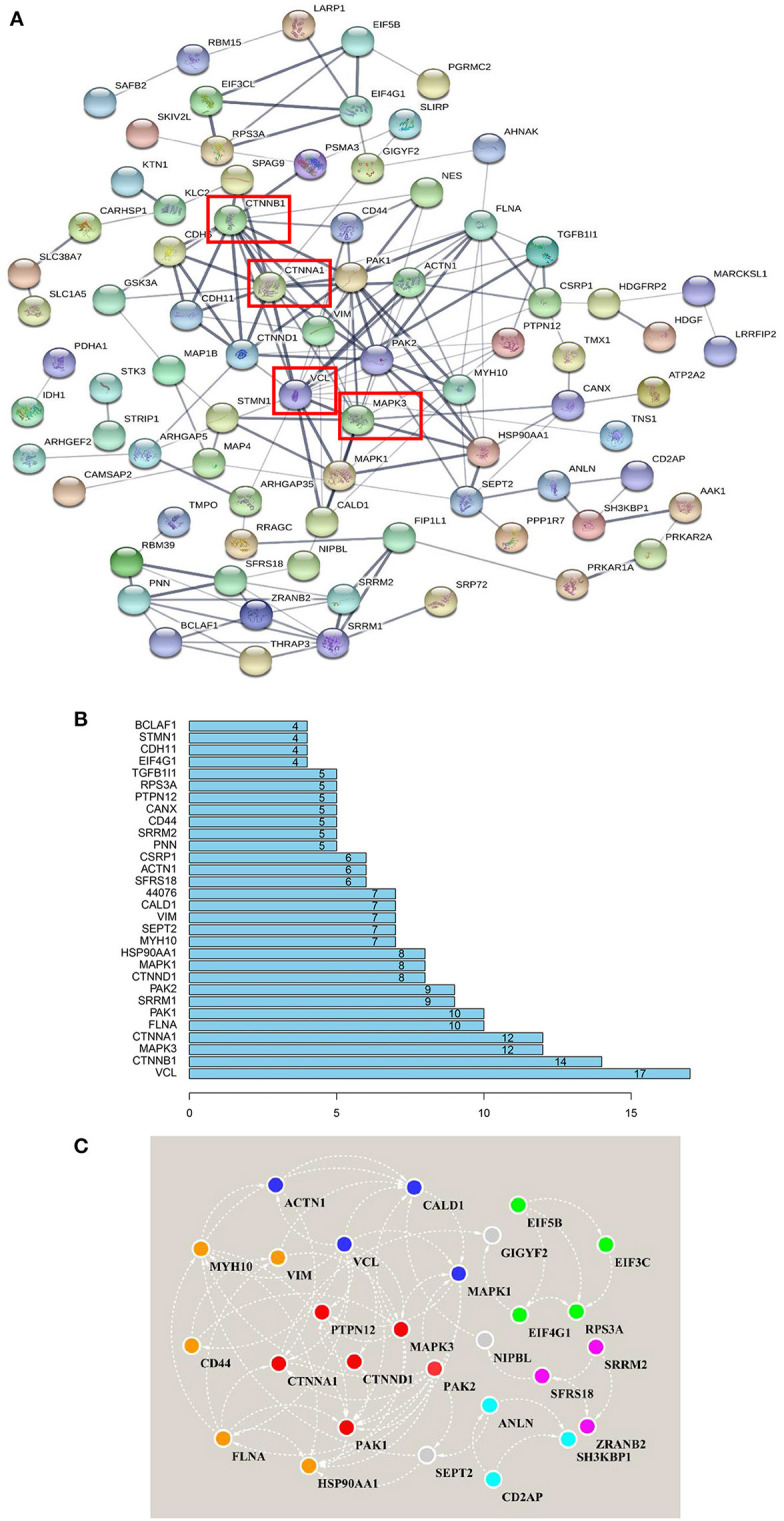
Interaction network analysis, based on the phosphorylated proteins positively associated with amino acid signals. **(A)** Proteins that were positively associated with all amino acid signals were input to the String database to perform network analysis. The string network indicated that VCL, CTNNB1, MAPK3, and CTNNA1 serve as the center of the entire network, with high credibility. Network nodes represent proteins, and network edges represent protein-protein associations. Empty nodes represent proteins of unknown 3D structure, whereas filled nodes represent proteins for which some 3D structure is known or predicted. The thicker the edges, the higher the confidence. **(B)** The distribution of protein nodes, showing the top 30 notes from **(A)**. **(C)** The raw data from the String interaction network analysis were input into Cytoscape software to predict subnetworks. Five subnetworks were identified, closely related to cytoskeletal regulation and protein synthesis. The same color nodes are used for the subnetwork.

As stated above, in Frame 4, the proteins for which phosphorylation abundance is downregulated after amino acid starvation and upregulated after stimulation with all amino acids may play pivotal roles in the intracellular signaling pathways induced by amino acid signals. Therefore, to explore the functional association networks associated with these proteins, we analyzed them using the String database. The results demonstrated that the identified proteins in Frame 4 featured extensive interactions. VCL, catenin beta 1 (CTNNB1), MAPK3, and catenin alpha 1 (CTNNA1) were associated with 17, 14, 12, and 12 nodes, respectively, serving as key factors in this network (Figures 6A,B). Subsequently, 5 subnetworks were identified by analyzing the network using the String database with Cytoscape software (Figure 6C), which were closely associated with cytoskeletal regulation and protein synthesis. This network revealed central proteins and phosphorylation transmission routes in the signal pathway networks induced by all amino acids.

### Motif Analysis of the Peptides Centered on Phosphosites That Were Correlated With Amino Acid Signals

**Figure 7:**
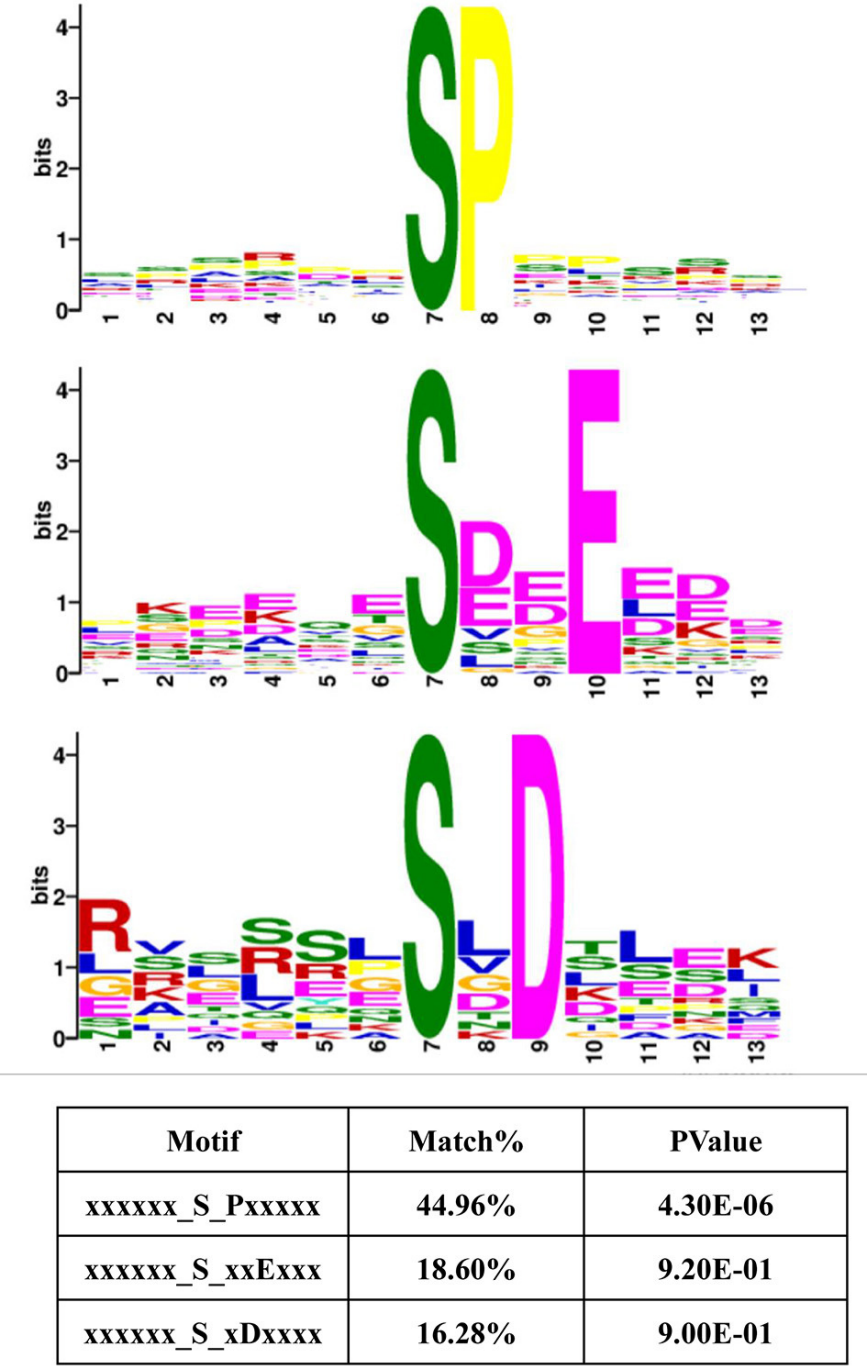
Motif analysis, based on the phosphorylated proteins that were positively associated with amino acid signals. Peptide sequences, featuring 13 amino acids, centered on the phosphorylated sites that were positively associated with amino acid signals were input into MoMo software from MEME. Three master motifs were identified: xxx_S_Pxx, xxx_S_xxE, and xxx_S_xDx.

Protein kinases typically display preferences for specific sequences in their substrates (38, 39). Therefore, these 13-amino acid peptide sequences, centered on the phosphosites in Frame 4, were input into MoMo software from MEME. This program can identify motifs representing amino acid preferences flanking phosphorylation sites. As a result, three master motifs were obtained, including xxx_S_Pxx, xxx_S_xxE and xxx_S_xDx. The proportions of these three motifs were 44.96, 18.60, and 16.28% of all peptide sequences centered on phosphosites in Frame 4, respectively (Figure 7). The results of motif analysis implied that specific kinase types are likely to play vital roles in the signaling pathways induced by all amino acids and that the interactions between kinases and subunits were highly conserved among different species.

### Analysis of the Potential Mechanism of Signal Transduction Induced by Amino Acid Signals in Goat Fetal Fibroblasts

In the present study, a total of 144 phosphosites on 102 proteins were identified for which the phosphorylation levels were positively correlated with amino acid signals, based on comparisons of quantitative data obtained from the phosphoproteomics analysis.

**Figure 8:**
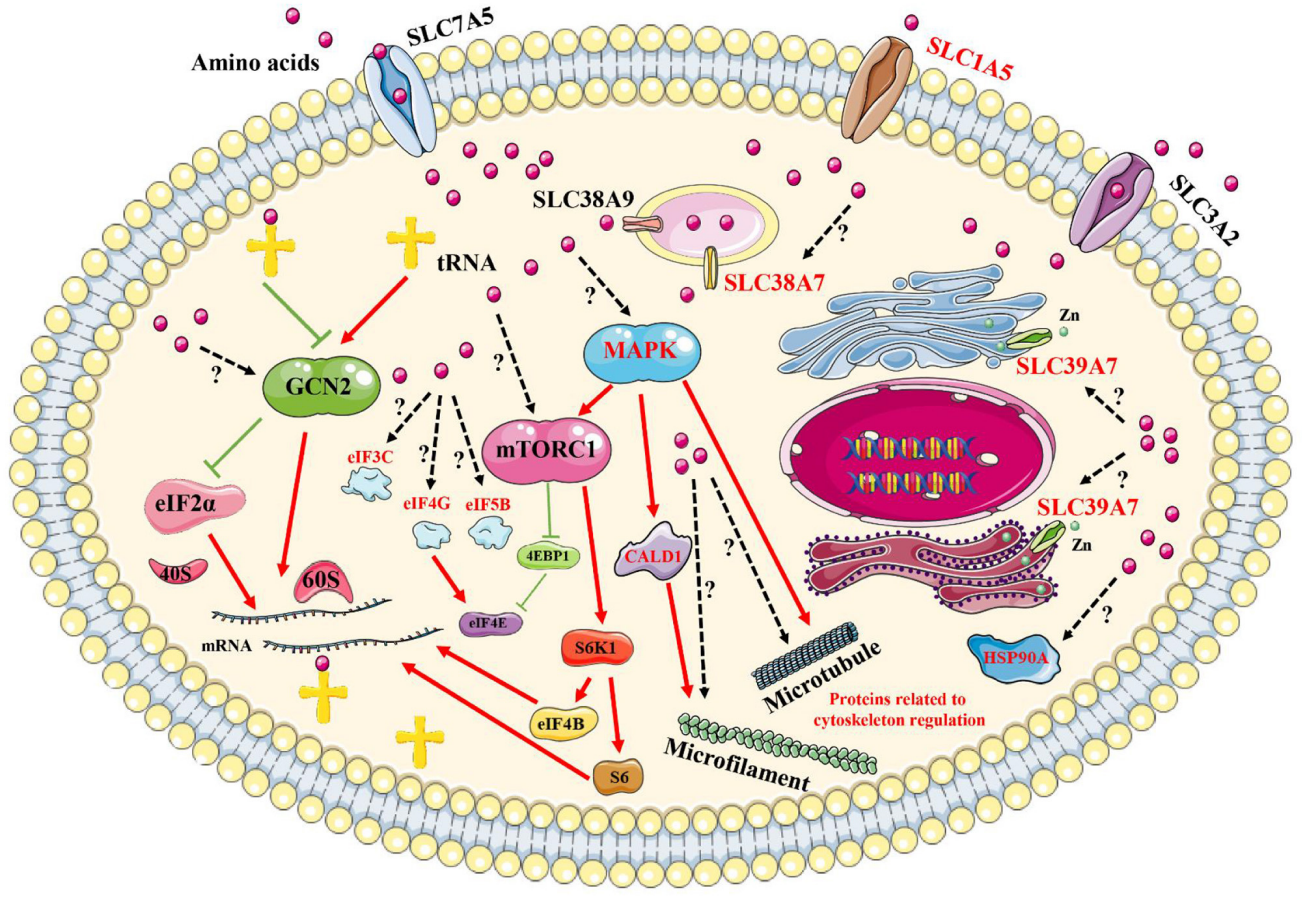
Brief model diagram showing the signaling pathways induced by amino acid signals. Amino acids may be sensed and transported by transporters on the plasma membrane and endomembrane system. Then, the signals are transmitted to the GCN2, mTORC1, and MAPK signaling pathways, which control various physiological and biochemical processes via the regulation of downstream effectors. Red marked proteins were identified in this study, with red lines representing activation, whereas green lines represent inhibition. Dotted lines and question marks represent related functions with unspecified mechanisms.

By performing comprehensive analyses of these results, in reference to previously published reports, some key proteins could be added to currently known signaling pathways, including the GCN2 pathway and the mTORC1 pathway, to generate a new network pathway model induced by amino acid signals (Figure 8). First, amino acid signals, both deficiencies and excesses, can be sensed and transported by membrane proteins, such as SLC39A7, SLC1A5, and SLC38A7. These signals are transmitted to a central intracellular pathway network through MAPK1/3, which mobilizes downstream effectors to initiate processes that allow for the cells to adapt to changes in their environments. These effectors may include the transcription factors eIF5B, eIF4G, and eIF3C, which regulate protein synthesis, and HSP90A, which controls specific protein degradation. The addition of new members to intracellular signaling pathways induced by amino acid signals provides new avenues for exploring the sensing mechanism associated with intracellular amino acid signals.

### Validation of Specific Phosphosites and Phosphoproteins Associated With Amino Acid Signals

**Figure 9:**
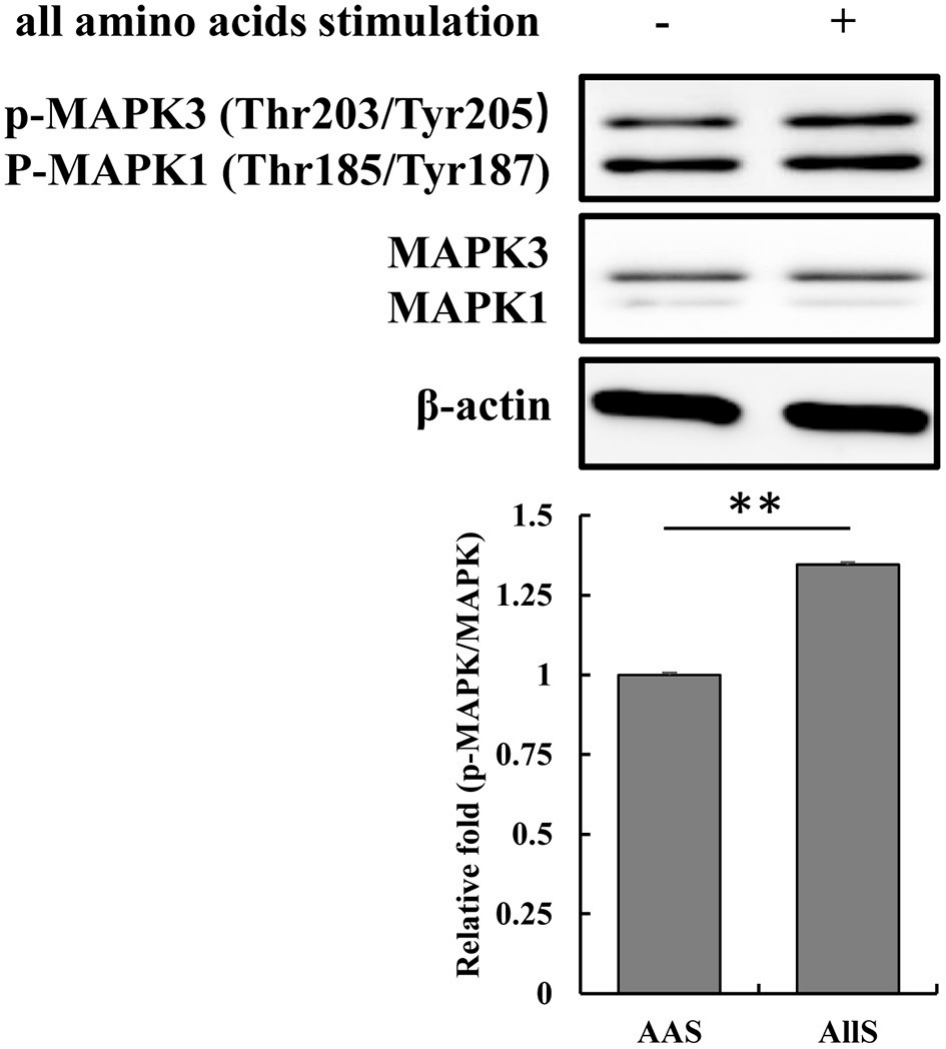
Validation of MAPK3 phosphorylation at threonine 203 and tyrosine 205 during amino acid starvation and restimulation with all amino acids. GFb cells were subjected to amino acid starvation for 1 h (AAS group), and restimulation with all amino acids for 1 h after amino acid starvation (AllS group). The cells were lysed, and the expression and phosphorylation levels of MAPK3 were analyzed by immunoblotting. Antibodies were derived from rabbits immunized with a synthetic phosphopeptide corresponding to the residues surrounding threonine 202 and tyrosine 204 of human MAPK3, which are identical to the residues surrounding threonine 203 and tyrosine 205 in goat MAPK3. All bands were quantified using Gel-Pro Analyzer 4.0 (^*^p < 0.05, ^**^p < 0.01, *n* = 3 experiments).

To validate the specific phosphosites and phosphoproteins that respond to amino acid signals, the threonine 203 and tyrosine 205 on MAPK3 were examined following amino acid signaling. The amino acid sequence of MAPK3 in goat features an extra alanine at position 7 compared with the human sequence, and the sequence of 100 amino acids centered on threonine 203 and tyrosine 205 for goat MAPK3 is identical to that centered on threonine 202 and tyrosine 204 in human MAPK3. Therefore, antibodies derived from the immunization of rabbits using a synthetic phosphopeptide corresponding to the residues surrounding threonine 202 and tyrosine 204 of the human MAPK3 sequence were used to detect the phosphorylation of threonine 203 and tyrosine 205 in goat MAPK3. The results demonstrated that the phosphorylation levels of MAPK at threonine 203 and tyrosine 205 increased following restimulation with all amino acids (Figure 9), which was consistent with the phosphoproteomics analysis results.

## Discussion

Arbas white Cashmere goats live in the desert and semi-desert grassland areas of western China. The amino acid composition of the local pasture is suitable for the growth of cashmere goats and may play an important role in the production and quality of both the meat and wool harvested from these animals. In the present study, we demonstrated that amino acid starvation and restimulation resulted in large-scale changes in the phosphorylation levels of many phosphorylation sites located on intracellular proteins in GFb cells. In this work, amino acid starvation for 1 h can lead to significant decrease in proteome phosphorylation in primary GFb cells, while a research on autophagy and phosphoproteomics did not demonstrated such a significant reduction in the total number of phosphoproteins after amino acid starvation for 30 min in MCF7 cells (40), which imply the difference in growth and metabolic pathway of cells and amino starvation time would affect phosphorylation level of proteome.

Phosphoproteomics profiling represents an effective method for the identification of vital proteins in cell signaling pathways. The identification of phosphorylation sites associated with phosphoproteins can help us better understand the molecular mechanisms involved in intracellular signal transduction pathways (41, 42). In the present study, the membrane proteins SLC39A7, SLC1A5, and SLC38A7 were identified in our screen. SLC39A7, SLC1A5, and SLC38A7 are three membrane transporters located on the endoplasmic reticulum (ER) and Golgi membranes, the plasma membrane, and the lysosome membrane, respectively. SLC39A7 controls zinc homeostasis within the cell and can activate multiple tyrosine kinases (43, 44). SLC1A5 is a neutral amino acid transporter that has been associated with greatly enhanced expression levels in stem cells and cancer cells to meet increased glutamine demand (45–47). SLC38A7 belongs to the SLC38 family, which also includes SL38A9, a known arginine sensor that regulates the mTORC1 signaling pathway on the lysosome membrane (48, 49). A latest research showed that SLC38A7 is responsible for transporting glutamine and asparagine and it is essential for lysosome degradating proteins to restore cytosolic amino acids, implying that SLC38A7 is closely related to autophagy (50). So far, few research have explored the role of phosphorylation in SLC39A7, SLC1A5, and SLC38A7, and phosphorylation may modulate transporting activity in these three membrane proteins, just as same as the Na^+^/K^+^-ATPases in which phosphorylation regulate pumping activity by altering chemical conformation. the phosphorylation of serine 275 and serine 276 on SLC39A7, serine 495 on SLC1A5, and serine 30 on SLC38A7 shown in our study would provide a foundation for revealing the mechanism of amino acids and other ions transport in cells.

There is no doubt that amino acid starvation, followed by restimulation with amino acids, will affect protein synthesis processes (51, 52). In this study, eIF5B, eIF4G, and eIF3C, three vital translation initiation factors, were also identified as being affected by changes in amino acid signaling. eIF5B is a critical factor that controls translation, from initiation to elongation, and may serve as a survival switch that regulates apoptosis (53, 54). Competing with 4EBPs, eIF4G interacts with eIF4E to facilitate the assembly of eIF4E and eIF4A into eIF4F, which regulates translation initiation, and 4EBP1 is a downstream effector of mTORC1 (55–57). Stimulation with hormones or mitogenic factors is thought to result in mTOR interacting directly with the eIF3 complex, causing the dissociation and phosphorylation of S6K1 (58). However, the function of eIF3C has not yet been fully explained. In the present study, we found that the phosphorylation levels of serine 216 on eIF5B, serine 1194 on eIF4G, and serine 39 on eIF3C varied greatly in response to amino acid starvation and restimulation. Those meaningful proteins have been added to the intracellular signaling pathway networks known to be induced by amino acid signals (Figure 8). These data provided evidence to support further in-depth research examining the control of protein synthesis by translation initiation factors in response to amino acid signals.

The MAPK signaling pathway is generally thought to sense growth factors and stress to control multiple physiological processes, and few studies have focused on the mechanisms through which the MAPK signaling pathway is induced in response to amino acid signals (59). In the present study, comprehensive functional analyses, based on identified KEGG pathways and interaction networks, were used to identify signaling pathways that were activated in response to amino acid starvation and restimulation. The MAPK signaling pathway was identified and predicted to play an extremely important role. In particular, MAPK1/3, also known as extracellular signal-related kinase (ERK)1/2, appears to represent the center of the entire network. Recently, tuberous sclerosis complex 2 (TSC2) was found to participate in the process through which mTORC1 responds to amino acid starvation, localizing to the lysosome to inactivate Rheb (60–62), and ERK is known to be an upstream negative regulator of TSC2 (63). MAPK3 phosphorylation at threonine 203 and tyrosine 205 was also found to increase after amino acid restimulation (Figure 9).

Because protein kinases display preferences for specific primary sequence in substrates (38, 39), motif analysis was performed on the peptides centered at those phosphosites that were identified to be positively regulated by signaling with all amino acids in our study. **Three** master motifs xxx_S_Pxx, xxx_S_xxE, and xxx_S_xDx were identified (Figure 7). The primary motif xxx_S_Pxx is the sequence associated with MAPK1/3 substrates (38), which is consistent with the results of the interaction network analysis, which showed that MAPK1/3 acts on a wide range of protein substrates, suggesting that the phosphorylation of these proteins may be positively regulated by amino acid signals. In this study, the response of the MAPK pathway to amino acid signals was as strong as the response of the mTORC1 pathway (Figures 2, 9). Thus, MAPK1/3 may be a potential mediator of the signaling pathway response to amino acid signals. Besides, Protein kinase CK2 (Casein kinase 2) has extensive function in cellular processes, and many cation and anion channels can be regulated by CK2 via direct or indirect phosphorylation (64). Coincidentally, motif xxx_S_xxE and xxx_S_xDx are shown as CK2 substrate sequences, implying that CK2 may be responsible for the regulation of amino acid transport (65).

In summary, this study has provided evidence to suggest that amino acid deficiency induces autophagy and that the mTORC1/ULK1 pathway responds to amino acid signals in Cashmere goat fetal fibroblasts. A total of 144 sites on 102 proteins were identified, associated with changes in phosphorylation levels in response to amino acid signals based on the results of phosphoproteomics analysis. The MAPK signaling pathway was identified as playing a potentially important role based on comprehensive function analyses, including KEGG pathway and interaction network analyses, and MAPK1/3 may serve as the center of the entire network. Furthermore, three membrane proteins, SLC39A7, SLC1A5, and SLC38A7, and three translation initiation factors, eIF5B, eIF4G, and eIF3C, were identified as proteins with phosphorylation levels that were positively regulated by all amino acid signaling. These pivotal proteins associated with amino acid signaling were added to the currently known amino acid signaling pathways to generate a novel diagram. The results of this study provided new insight into the precise mechanisms through which amino acid signals are sensed in Cashmere goat fetal fibroblasts.

## Data Availability Statement

The datasets presented in this study can be found in online repositories. The names of the repository/repositories and accession number(s) can be found below: http://proteomecentral.proteomexchange.org/, PXD025488.

## Ethics Statement

The animal study was reviewed and approved by the Inner Mongolia University Animal Care and Use Committee.

## Author Contributions

The work was mainly designed and conceived by YW, XG, and ZW. Experiments were performed by XZ and HS. Experimental data were collected and analyzed by LW and RY. The manuscript was mainly written by XZ and revised by LB and YM. All the authors contributed, read, and approved the final manuscript.

## Conflict of Interest

The authors declare that the research was conducted in the absence of any commercial or financial relationships that could be construed as a potential conflict of interest.

## Publisher's Note

All claims expressed in this article are solely those of the authors and do not necessarily represent those of their affiliated organizations, or those of the publisher, the editors and the reviewers. Any product that may be evaluated in this article, or claim that may be made by its manufacturer, is not guaranteed or endorsed by the publisher.
