# Supplementary material for: Phosphoproteomics Analysis Reveals a Pivotal Mechanism Related to Amino Acid Signals in Goat Fetal Fibroblast
Source: Front Vet Sci. 2021 Aug 3;8:685548. doi: 10.3389/fvets.2021.685548 (PMC8370256; doi:10.3389/fvets.2021.685548)
Supplement: Supplementary file 1 [file Table_1.DOCX]

**Table S1 Relative quantitative data of phosphosites responding to all amino acids signal**

| Protein names | Protein ID | Phosphosites | log_10_(FC)  (AAS group vs. SS group) | log_10_(FC)  (AllS group vs. AAS group) |
| --- | --- | --- | --- | --- |
| HSP90A | chx:100860851 | Ser252 | -2.03 | 1.37 |
| TGFB1I1 | chx:100861032 | Ser167 | -1.81 | 1.07 |
| PDXDC1 | chx:102176932 | Thr688 | -1.79 | 1.65 |
| BCLAF1 | chx:102180497 | Ser512 | -1.76 | 1.42 |
| TGFB1I1 | chx:100861032 | Ser171 | -1.71 | 1.03 |
| SLC1A5 | chx:102176550 | Ser495 | -1.69 | 0.58 |
| AAK1 | chx:102187599 | Ser646 | -1.66 | 1.62 |
| MAP1 | chx:102176074 | Ser1782 | -1.64 | 1.52 |
| STMN1 | chx:102173139 | Ser25 | -1.62 | 2.05 |
| MAP1 | chx:102176074 | Ser1779 | -1.42 | 1.22 |
| SAFB2 | chx:102183590 | Ser342 | -1.40 | 0.98 |
| TGFB1I1 | chx:100861032 | Ser173 | -1.36 | 2.17 |
| MYH10 | chx:102177371 | Ser1987 | -1.29 | 1.22 |
| AIM1 | chx:102180395 | Ser1499 | -1.26 | 1.16 |
| CCDC6 | chx:102172968 | Ser246 | -1.25 | 1.38 |
| CCDC6 | chx:102172968 | Ser242 | -1.25 | 1.16 |
| MAPK1 | chx:102186040 | Tyr187 | -1.20 | 1.11 |
| SH3KBP1 | chx:102186673 | Ser209 | -1.19 | 0.97 |
| SLIRP | chx:102173011 | Ser105 | -1.15 | 0.34 |
| HDGFL2 | chx:102172140 | Ser667 | -1.14 | 0.80 |
| SEPT2 | chx:102189730 | Ser218 | -1.14 | 0.96 |
| HSP90A | chx:100860851 | Ser231 | -1.12 | 1.11 |
| FLNA | chx:102176601 | Ser969 | -1.11 | 1.13 |
| SLC39A7 | chx:102182003 | Ser275 | -1.06 | 0.84 |
| SLC39A7 | chx:102182003 | Ser276 | -1.06 | 0.84 |
| TNS1 | chx:102188266 | Ser1404 | -1.03 | 0.49 |
| VCL | chx:102188740 | Ser290 | -1.03 | 0.45 |
| KTN1 | chx:102187323 | Ser75 | -1.00 | 0.76 |
| STMN1 | chx:102173139 | Ser16 | -0.98 | 1.36 |
| MAP1 | chx:102176074 | Ser1949 | -0.98 | 0.86 |
| NRBP1 | chx:102176972 | Thr8 | -0.96 | 0.77 |
| GSK3A | chx:102178478 | Ser278 | -0.96 | 0.34 |
| MAP1 | chx:102176074 | Ser1797 | -0.95 | 1.26 |
| PRKAR2A | chx:102180355 | Ser96 | -0.93 | 0.74 |
| MAPK3 | chx:102188824 | Tyr205 | -0.91 | 0.62 |
| CDH11 | chx:102173493 | Ser788 | -0.90 | 0.45 |
| TCOF1 | chx:102174025 | Ser1198 | -0.89 | 0.58 |
| SRP72 | chx:102177423 | Ser621 | -0.89 | 0.85 |
| CTNND1 | chx:102183255 | Ser349 | -0.87 | 0.44 |
| SRRM2 | chx:102185967 | Ser1242 | -0.87 | 0.33 |
| PDHA1 | chx:102168710 | Ser293 | -0.86 | 0.79 |
| SPAG9 | chx:102177747 | Ser730 | -0.85 | 0.65 |
| SPAG9 | chx:102177747 | Ser733 | -0.85 | 0.65 |
| MYADM | chx:102170725 | Ser18 | -0.83 | 0.64 |
| PPP1R7 | chx:102188014 | Ser57 | -0.83 | 0.70 |
| PPP1R7 | chx:102188014 | Ser60 | -0.83 | 0.70 |
| MAP4 | chx:102171384 | Ser282 | -0.83 | 0.68 |
| ARHGEF2 | chx:102181739 | Ser381 | -0.82 | 0.44 |
| CD44 | chx:102182426 | Ser679 | -0.82 | 0.33 |
| P97BCNT | chx:100860830 | Ser68 | -0.82 | 0.71 |
| CANX | chx:102173831 | Ser555 | -0.81 | 0.55 |
| TCOF1 | chx:102174025 | Ser1227 | -0.79 | 0.56 |
| MAP1 | chx:102176074 | Ser1785 | -0.78 | 0.67 |
| FAM129A | chx:102187885 | Ser708 | -0.77 | 0.73 |
| PDHA1 | chx:102168710 | Ser300 | -0.77 | 0.55 |
| TMPO | chx:102185249 | Thr160 | -0.77 | 0.45 |
| ACTN1 | chx:102176966 | Ser140 | -0.76 | 0.66 |
| PGRMC2 | chx:102173114 | Thr235 | -0.76 | 0.61 |
| CARHSP1 | chx:102188728 | Ser41 | -0.75 | 0.95 |
| THRAP3 | chx:102177859 | Ser686 | -0.74 | 0.55 |
| RRAGC | chx:102171835 | Ser95 | -0.74 | 0.44 |
| SRP72 | chx:102177423 | Ser625 | -0.74 | 0.46 |
| CSRP1 | chx:102184138 | Ser192 | -0.74 | 0.64 |
| CDH6 | chx:102182729 | Ser786 | -0.73 | 0.35 |
| PRKAR1A | chx:102178945 | Ser82 | -0.73 | 0.67 |
| PTPN12 | chx:102187831 | Ser435 | -0.72 | 0.69 |
| SRRM2 | chx:102185967 | Ser1330 | -0.72 | 0.73 |
| LRRFIP2 | chx:102180895 | Ser328 | -0.72 | 0.59 |
| MAPK3 | chx:102188824 | Thr203 | -0.71 | 0.86 |
| PSMA3 | chx:102168353 | Ser250 | -0.70 | 0.39 |
| MAP4 | chx:102171384 | Ser1879 | -0.70 | 0.85 |
| CARHSP1 | chx:102188728 | Ser32 | -0.69 | 0.93 |
| CARHSP1 | chx:102188728 | Ser30 | -0.69 | 0.91 |
| MAP4 | chx:102171384 | Ser415 | -0.69 | 1.21 |
| OSBPL11 | chx:102169629 | Ser190 | -0.69 | 0.35 |
| VIM | chx:102182515 | Ser325 | -0.68 | 0.48 |
| FAM129A | chx:102187885 | Ser704 | -0.68 | 0.31 |
| RBM39 | chx:102175418 | Ser136 | -0.68 | 0.52 |
| ARHGAP35 | chx:102177274 | Ser1180 | -0.67 | 0.33 |
| AKAP12 | chx:102172157 | Ser572 | -0.67 | 0.51 |
| STK3 | chx:102191439 | Ser316 | -0.66 | 0.48 |
| VCL | chx:102188740 | Ser795 | -0.66 | 0.35 |
| SRRM2 | chx:102185967 | Ser1173 | -0.66 | 0.73 |
| CALD1 | chx:102188560 | Ser784 | -0.66 | 0.99 |
| NDRG1 | chx:102168471 | Ser330 | -0.66 | 0.91 |
| MAP1 | chx:102176074 | Ser2034 | -0.65 | 0.50 |
| USP8 | chx:102174573 | Ser685 | -0.65 | 0.47 |
| FIP1L1 | chx:102171773 | Ser466 | -0.65 | 0.54 |
| HDGF | chx:102172855 | Ser133 | -0.65 | 0.42 |
| EIF4G | chx:102183267 | Ser1194 | -0.65 | 0.72 |
| ISYNA1 | chx:102191513 | Ser523 | -0.65 | 0.39 |
| ATP2A2 | chx:102174960 | Ser663 | -0.64 | 0.35 |
| RPS3A | chx:102189233 | Ser263 | -0.64 | 0.34 |
| SRRM1 | chx:102175909 | Ser883 | -0.64 | 0.41 |
| BCLAF1 | chx:102180497 | Ser285 | -0.63 | 0.63 |
| BCLAF1 | chx:102180497 | Ser290 | -0.63 | 0.41 |
| ZRANB2 | chx:102171565 | Ser153 | -0.63 | 0.38 |
| TNS1 | chx:102188266 | Thr1305 | -0.62 | 0.60 |
| HDGF | chx:102172855 | Ser132 | -0.62 | 0.39 |
| TNS1 | chx:102188266 | Ser1337 | -0.62 | 0.47 |
| XPC | chx:102170831 | Ser89 | -0.62 | 0.55 |
| SRRM2 | chx:102185967 | Ser2429 | -0.61 | 0.44 |
| STMN1 | chx:102173139 | Ser38 | -0.61 | 1.54 |
| CTNNA1 | chx:102189579 | Ser652 | -0.60 | 0.39 |
| LARP1 | chx:102176233 | Thr529 | -0.59 | 0.36 |
| STRIP1 | chx:102187729 | Ser335 | -0.59 | 0.37 |
| IDH1 | chx:102190002 | Ser162 | -0.59 | 0.43 |
| THRAP3 | chx:102177859 | Ser254 | -0.58 | 0.85 |
| EIF5B | chx:102178080 | Ser216 | -0.58 | 0.42 |
| VCL | chx:102188740 | Ser721 | -0.57 | 0.63 |
| ANLN | chx:102178507 | Ser187 | -0.57 | 0.38 |
| TMX1 | chx:102187493 | Ser245 | -0.57 | 0.72 |
| CTNNB1 | chx:102191742 | Ser191 | -0.57 | 0.33 |
| MARCKSL1 | chx:108637962 | Ser104 | -0.56 | 0.35 |
| FLNA | chx:102176601 | Ser2153 | -0.56 | 0.46 |
| KLC2 | chx:102174462 | Ser610 | -0.56 | 0.32 |
| CTNNB1 | chx:102191742 | Ser552 | -0.56 | 0.62 |
| PNISR | chx:102186767 | Ser211 | -0.55 | 0.30 |
| ARHGAP5 | chx:102178574 | Ser1219 | -0.55 | 0.46 |
| RBM15 | chx:102186923 | Ser701 | -0.54 | 0.44 |
| PAK1 | chx:102180827 | Ser144 | -0.52 | 0.31 |
| SLC38A7 | chx:102177558 | Ser30 | -0.52 | 0.32 |
| TNS1 | chx:102188266 | Ser1233 | -0.51 | 0.47 |
| CHAMP1 | chx:102186606 | Ser216 | -0.51 | 0.47 |
| EIF3C | chx:102182187 | Ser39 | -0.51 | 0.33 |
| GFPT1 | chx:102175034 | Ser243 | -0.50 | 0.31 |
| SAMHD1 | chx:102178551 | Ser266 | -0.49 | 0.36 |
| AHNAK | chx:102181273 | Ser178 | -0.49 | 0.60 |
| SRRM1 | chx:102175909 | Ser260 | -0.49 | 0.37 |
| MAP1 | chx:102176074 | Thr1788 | -0.48 | 0.32 |
| CAMSAP2 | chx:102172833 | Ser463 | -0.48 | 0.39 |
| VIRMA | chx:102186498 | Ser1579 | -0.48 | 0.36 |
| GIGYF2 | chx:102188464 | Ser26 | -0.47 | 0.71 |
| VIM | chx:102182515 | Ser430 | -0.46 | 0.63 |
| CD2AP | chx:102184676 | Ser461 | -0.46 | 0.41 |
| NES | chx:102173142 | Ser471 | -0.46 | 0.33 |
| BCLAF1 | chx:102180497 | Ser658 | -0.43 | 0.33 |
| SKIV2L | chx:102178026 | Ser256 | -0.42 | 0.32 |
| NIPBL | chx:102171102 | Ser2658 | -0.42 | 0.33 |
| THRAP3 | chx:102177859 | Ser249 | -0.41 | 0.73 |
| AP3B1 | chx:102173571 | Ser276 | -0.41 | 0.43 |
| PNN | chx:102187538 | Ser100 | -0.38 | 0.70 |
| RANBP3 | chx:102191605 | Ser348 | -0.38 | 0.42 |
| PAK2 | chx:102176919 | Ser141 | -0.32 | 0.61 |
